# Effects of early aquatic exercise intervention on trunk strength and functional recovery of patients with lumbar fusion: a randomized controlled trial

**DOI:** 10.1038/s41598-023-37237-3

**Published:** 2023-07-03

**Authors:** An-Hua Huang, Wen-Hsiang Chou, Wendy Tzyy-Jiuan Wang, Wen-Yin Chen, Yi-Fen Shih

**Affiliations:** 1 Department of Physical Therapy and Assistive Technology, National Yang Ming Chiao Tung University, 155, Li-Nong St Section 2, Pei-Tou District, Taipei, 112 Taiwan; 2 Department of Rehabilitation, Cheng Hsin General Hospital, Taipei, Taiwan; 3 Department of Orthopedics, Cheng Hsin General Hospital, Taipei, Taiwan

**Keywords:** Rehabilitation, Randomized controlled trials

## Abstract

This study investigated the effectiveness of an early aquatic exercise program on trunk muscle function and functional recovery of patients with lumbar fusion. Twenty-eight subjects were divided into two equal groups. Patients in the aquatic group performed two 60-min aquatic exercise sessions and three 60-min home exercise sessions per week for 6 weeks, whereas those in the control group performed five sessions of 60-min home exercises per week for 6 weeks. The primary outcomes were the Numerical Pain Rating Scale (NPRS) and Oswestry Disability Index (ODI), and the secondary outcomes were Timed Up and Go Test (TUGT), trunk flexor and extensor muscle strength, lumbopelvic stability, and lumbar multifidus muscle thickness measured pre- and post-intervention. Compared with participants in the control group, those in the experimental group showed significant improvement in NPRS, ODI, trunk extensor strength, lumbopelvic control, lumbar multifidus muscle thickness, and relative change in multifidus muscle thickness (significant time by group interactions, P < 0.05). Participants in both groups showed significant time effects (P < 0.001) for TUGT and trunk flexor strength outcome. Aquatic exercise combined with home exercise was superior to home exercise alone in reducing pain, disability and improving muscle strength, lumbopelvic stability, and lumbar multifidus muscle thickness.

## Introduction

Lumbar fusion is a common spine surgery often performed to relieve symptoms related to spinal degenerative changes, stenosis, and spondylolisthesis^1,2^. However, some patients complain of residual symptoms after surgery, with a 10–40% rate of unsatisfactory results^3^. Residual symptoms include persistent pain, motor deficits, decreased functional status, and inability to return to work. A decrease in strength and atrophy of the back muscles after surgery might contribute to these postsurgical complications^4^. Postoperative interventions such as muscle strengthening and functional training are typically commenced 3 months after surgery. Beginning muscle strengthening as early as 6 weeks after surgery has been suggested, and researchers have reported that early conditioning of the trunk muscles would increase muscle strength, reduce atrophy, and thus minimize disability and improve the quality of life of the patient^5^.

Exercise in water, compared with that on land, reduces load^6–8^. The level of trunk muscle activity recorded using surface electromyography (EMG) during activities performed in water has been reported to be less than 25% of maximal voluntary contraction (MVC)^9^. This level of muscle activation is lower than the reported threshold of 40% MVC at which there is an increased risk of joint pain or injury to the spine^10^. Water immersion reduces loading of the spine because of buoyancy and allows for movements that are normally difficult to execute on land^11,12^. Therefore, aquatic trunk exercise is considered safe for those who have undergone lumbar spine surgery. Previous studies have reported that aquatic exercise programs resulted in improved strength and quality of life and reduced pain and disability in patients with low back pain (LBP)^13,14^. Similar effects of aquatic exercise intervention were also observed in patients after total knee and hip replacement and anterior cruciate ligament reconstruction^15–17^. However, no study has investigated the effect of aquatic exercise in patients after lumbar fusion surgery.

Therefore, through this study, we aimed to examine the effects of an early aquatic rehabilitation program on pain, disability, trunk muscle strength, lumbopelvic stability, and lumbar multifidus (LM) muscle thickness following lumbar spine fusion. We hypothesized that a 6-week aquatic rehabilitation program would significantly improve trunk muscle strength, lumbopelvic stability, and LM muscle thickness and reduce pain and disability in patients who have undergone lumbar fusion.

## Results

**Figure 1:**
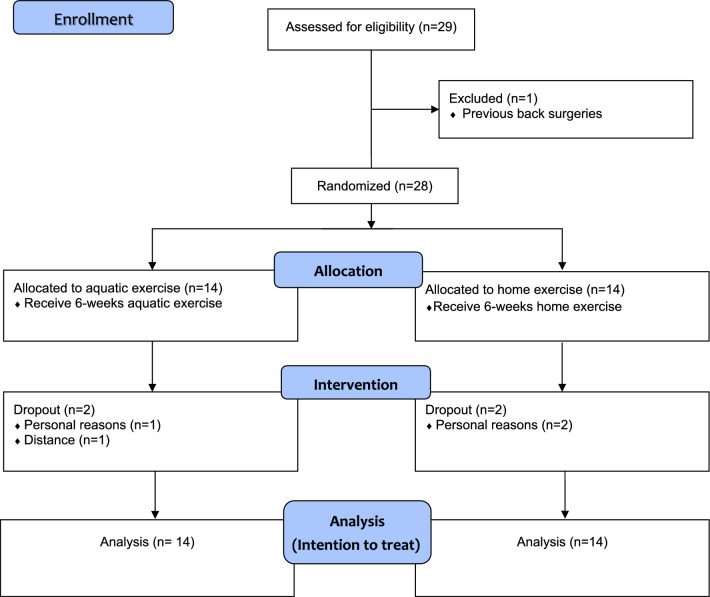
Flow chart of the study.

In total, 28 participants who met the inclusion criteria were recruited and randomized into either group (14 participants per group). Four participants (two from the experimental group and two from the control group) dropped out from the trial, and 24 participants (experimental group, n = 12; control group, n = 12) completed the study. Our last observation results were carried forward in the dropped out data for the intention-to-treat analysis. Figure 1 shows the flowchart of recruitment of study participants, including reasons for their dropout. The demographic data of the participants are shown in Table 1. No significant between-group difference was observed in the participants at baseline.

**Table 1:** Comparisons of the demographic data of the experimental and control group, using the independent *t* test for continuous data (age, height, weight and BMI) and chi-square tests for categorical data (gender).

	Experimental group (n = 14)	Control group (n = 14)	P value
	Mean (SD)	Mean (SD)	
Age (years)	49.64 (10.18)	54.36 (8.32)	0.19
Gender (male/female)	8/6	7/7	0.37
Height (cm)	167.64 (7.19)	162.86 (7.54)	0.09
Weight (kg)	70.75 (12.55)	68 (15.65)	0.61
BMI (kg/m^2^)	24.43 (3.15)	25.95 (3.98)	0.27

*SD* standard deviation.

Table 2 summarizes the comparisons between the two groups before and after the intervention. After 6 weeks of training, a significant group-by-time interaction was observed with regard to NPRS (*P* = 0.003) and ODI (*P* = 0.01); the mean difference between the groups was − 1.5 points (95% CI 0.2, − 2.8) and − 5.73 points (95% CI − 0.56, 17.52) in the NPRS and ODI after intervention. The post hoc tests indicated that only the experimental group experienced a significant decrease in pain by an additional − 1.93 points (95% CI − 3.08, − 0.78; *P* = 0.002), whereas both groups reported significantly decreased ODI scores (experimental group: *P* < 0.001, control group: *P* = 0.028).

**Table 2:** Comparisons between the experimental and control groups before and after the intervention, using the two-way repeated measures analysis of variance (ANOVA) with the Bonferroni corrections as the post hoc test.

Outcome	Experimental group (n = 14)	Control group (n = 14)	Mean difference between groups (95% CI)	p value	Group by time effect
NPRS					
Baseline	3.21 ± 2.72	2.14 ± 1.1	1 (− 0.66 to 2.66)	0.221	0.003*
Post test	1.29 ± 0.99^†^	2.78 ± 2.15	− 1.5 (0.2 to − 2.8)	0.02	
ODI					
Baseline	42.24 ± 13.41	34.25 ± 14.89	7.99 (− 3.02 to 19)	0.148	0.01*
Post test	20.43 ± 18.15^†^	26.16 ± 11.45^†^	− 5.73 (− 0.56 to 17.52)	0.327	
TUGT					
Baseline	10.79 ± 1.96	10.42 ± 2.61	0.34 (− 1.45 to 2.13)	0.7	0.53
Post test	8.96 ± 2.03^†^	9.37 ± 1.92^†^	− 0.41 (− 1.13 to 1.95)	0.747	
Trunk flexor					
Baseline	20.6 ± 11.04	14.75 ± 9.06	5.85 (− 2 to 13.7)	0.14	0.183
Post test	25.9 ± 14.52^†^	19.35 ± 9.17^†^	9.41 (− 0.11 to 18.93)	0.53	
Trunk extensor					
Baseline	22.73 ± 11.82	19.49 ± 11.46	3.24 (− 5.8 to 12.29)	0.468	0.013*
Post test	32.51 ± 20.9^†^	22.56 ± 10.07	12.8 (0.6 to 25)	0.04	
Lumbopelvic stability					
Baseline	8.49 ± 7.99	8.83 ± 6.63	− 0.34 (− 6.04 to 5.36)	0.904	0.018*
Post test	25.24 ± 19.5^†^	12.94 ± 14.37	13.02 (− 0.05 to 26.08)	0.051	
LM thickness rest					
Baseline	28.68 ± 4.62	29 ± 7.0	0.6 (− 0.43 to 0.55)	0.805	0.001*
Post test	32.95 ± 6.60^†^	28.61 ± 5.57	4.55 (− 0.24 to 9.33)	0.062	
Relative change in the LM thickness %					
Baseline	6.33 ± 5.49	7.00 ± 5.83	− 0.68 (− 5.08 to 3.71)	0.752	0.044*
Post test	8.59 ± 3.49^†^	6.05 ± 3.98	0.98 (− 2.78 to 4.75)	0.096	

Data are represented as mean ± standard deviation and mean difference (95% confidence interval).

*NPRS* numerical pain rating scale, *ODI* Oswestry Disability Index, *TUGT* timed up and go test, *LM* lumbar multifidus.

*Significant group by time interaction, *P* < 0.05.

^†^Significant difference between baseline and post-test values using Bonferroni corrections, *P* < 0.05.

Six weeks of training did not show a statistically significant group-by-time interaction in the TUGT, with the mean difference between interventions was 0.41 s (95% CI − 1.13, 1.95; *P* = 0.53). Both groups demonstrated significant within-group improvement (time effect: p < 0.001; experimental group: − 1.8 s, 95% CI − 2.68, − 0.9, *P* < 0.001; control group: − 1.05 s, 95% CI − 1.05, 0.44, *P* = 0.024).

Both groups showed significant improvement in trunk flexor strength (time effect: p < 0.001; experimental group: 8.16 Kgw, 95% CI 4.37, 11.95, *P* < 0.001; control group: 4.6 Kgw, 95% CI 0.81, 8.38, *P* = 0.019), whereas only the experimental group showed a significantly improved trunk extensor strength of 12.64 Kgw (a significant time by group effect, 95% CI 0.6, 25; *P* = 0.013).

A significant group-by-time interaction was observed for lumbopelvic stability (95% CI − 0.05 to 26.08; *P* = 0.018) and the post hoc tests indicated that only the experimental group experienced a significant improvement in testing pressure of 17.47 mmHg (95% CI 9.77, 25.17, *P* < 0.001) for the lumbopelvic stability test. The analysis also revealed a significant group-by-time interaction (95% CI − 2.78, 4.75; *P* = 0.044) for the relative change in the LM thickness. The post hoc tests indicated that only the experimental group exhibited significantly increased LM muscle relative change by an additional 2.28 mm (95% CI 0.04, 4.52; *P* = 0.046).

## Discussion

To the best of our knowledge, this is the first study to investigate the effects of an early aquatic rehabilitation program on pain, disability, and trunk muscle function following lumbar spine fusion. Our data supported most of the hypotheses, with the 6-week aquatic rehabilitation program significantly improving trunk muscle strength, lumbopelvic stability, and LM muscle thickness, and reducing pain and disability in patients after lumbar fusion compared with the home exercise program.

Significant improvement in pain score was observed only in the experimental group. Although no research has been performed on the effects of aquatic exercise for lumbar fusion, similar pain relief effects of aquatic exercise intervention were observed in patients after total knee and hip replacement^15,17^, in research on LBP^18^, and in another meta-analysis^19^. Studies have shown that the hydrostatic effect of water exercise can relieve pain by reducing peripheral edema and inhibiting the activity of the sympathetic nervous system^20^. Hence, we contend that aquatic exercise exerts a relaxation effect owing to water immersion, which effectively reduces the perception of pain^6^. In our study, we found no improvement in pain in the control group; however, previous studies have shown that lumbar stabilization exercises and strengthening of lumbar muscles reduced pain in patients with LBP^21–23^ and in those who had undergone lumbar fusion^5,24,25^. This discrepancy could be attributable to the fact that our control group performed only a home exercise program instead of a supervised exercise intervention.

In this study, both the aquatic rehabilitation program and home exercise program significantly improved the disability score, although better results were observed with aquatic exercise than with home exercise. We observed a 37.82% decrease in ODI in the experimental group, which was better than the previously reported data (18% and 36%)^21,24^. Dundar et al. randomly assigned 65 patients with chronic LBP to a land-based or an aquatic-based program; the results showed that aquatic exercises resulted in better improvement of disability than the land-based exercises did^12^; this may be attributable to the fact that water buoyancy reduces joint compressive forces during exercise^8^, thereby improving the confidence of patients and increasing their willingness to move their spine. The improved ODI scores in the home exercise group may be attributable to the training received in performing back exercises; these findings are consistent with studies that reported that back exercise training improved the ODI scores of patients after lumbar fusion surgery^21,26^.

The TUGT is an assessment tool that reflects the lower extremity function and the static and dynamic balance of patients who undergo lumbar fusion. This assessment also evaluates movements necessary for ambulation in daily life^27^. Therefore, our findings suggested that both exercise programs may be helpful for improving static and dynamic balance on land among patients who undergo lumbar fusion. Although our experimental group performed 6-week aquatic exercises together with a home program, the program may have been insufficient to confer an additional benefit on the patients. Another study reported that aquatic exercise training using water-resistance equipment improved balance and walking ability among elderly individuals^28^. Our program did not comprise resistance walking training in the water; hence, no significant difference was observed between the balance and walking ability of the two groups in our study.

The experimental group exhibited significantly improved trunk extensor strength. After surgery, the trunk extensor muscle is damaged, and the patient is typically afraid to do land exercises. However, the buoyancy of water can reduce the body’s weight and load on the muscle. This can help overcome the sense of fear and pain experienced by patients that typically lead to reduced movement, thereby enhancing their willingness to move in the water. In addition, an aquatic environment may allow patients to perform exercises to reestablish motor control without exacerbating their symptoms^29^. An increase in trunk muscle strength after fusion surgery can help patients become more confident in using their back during daily activities and reduce their chances of developing disability, which is often caused by avoidance behavior. Although there was no significant between-group difference in trunk flexor strength improvement, both group showed increased trunk flexor strength after 6 weeks of exercise (a significant time effect). As trunk flexor muscles were not damaged during surgery, there was less concern of avoidance behavior, and both types of exercise showed significant effects on improving trunk flexor strength.

The lumbopelvic stability test revealed a significant intervention effect between patients in the experimental and control groups. Studies have shown that strengthening the lumbar stabilizer muscles such as transverse abdominis and multifidus can improve trunk control ability^30–32^. Bressel et al. and Psycharakis et al. used EMG to study trunk muscle activation during stability exercises in water and found that aquatic exercises are more beneficial for lower abdominal muscles (internal oblique/ transversus abdominis) and the LM muscles than for other trunk muscles^33,34^, as they effectively induce the contraction of lumbopelvic stabilizer muscles and the LM muscles in water. This evidence supports the effect of aquatic exercises on lumbopelvic stability.

A recent study, conducted in 2021, reported that hip abductor strengthening exercises can increase the size of LM muscles^35^. The movements in these exercises were similar to those in our exercise program. This might explain the significant improvements in the size of the LM muscles only in the experimental group. Bressel et al. indicated that hip abduction movement in water affects trunk balance, and that maintaining trunk balance in the water enables the greater activation of the LM muscle^36^. Although the LM is the most affected muscle during lumbar fusion and previous researchers have reported that lumbar surgery can be one of the most important causes of atrophy of the back muscle^37^, the LM muscle has a bipennate structure, with fibers crossing one, two, and even three vertebra levels; thus, even patients who undergo lumbar fusion surgery can train the upper and lower sections of the LM muscle to achieve the training effect.

Several limitations of this study may partially interfere with the results. First, as the study lacked a long-term follow-up, it is unclear whether the observed improvements could be maintained for a longer period of time. Second, the study was not blinded, and thus, the outcome could not entirely exclude bias in the measurement of results. Finally, although the exercise frequency of the participants in the control group was recorded, no therapist supervised their exercises, whereas the participants in the experimental group were supervised twice while they received their aquatic exercise training. Hence, it is unclear whether the positive effects are attributable to hydrotherapy or supervision.

## Methods

### Trial design

This was an experimental, randomized controlled trial. Participants were randomly allocated to an experimental group or a control group for 6 weeks of intervention. This study was approved by the Institutional Review Board of Cheng Hsin General Hospital (425-103-02) and registered on 13/11/2016 at Thai Clinical Trials (registration number TCTR20161113001) and consistent with the CONSORT checklist. All experiments were performed in compliance with applicable guidelines and regulations.

### Participants

Twenty-eight patients who underwent surgery with lumbar fusion and were referred from the Department of Orthopedics to the Department of Rehabilitation of the Cheng Hsin General Hospital for physiotherapy between October 2014 and December 2016 were recruited. Participants scheduled for first lumbar fusion surgery, those aged between 20 and 65 years, and those with unilateral leg symptoms prior to surgery were included. Those who had previously undergone back surgeries; those with severe cardiovascular disease, neurological disorders, or cognitive dysfunction; and pregnant patients were excluded. All participants signed written informed consent forms before participation. All data were collected at the Department of Rehabilitation in Cheng Hsin General Hospital.

### Sample size and randomization

The sample size was calculated using the G*Power software, with alpha = 0.05 and 80% power for the outcome of the Oswestry Disability Index (ODI) by using the formula proposed by Abbott^38^. A total of 12 individuals were selected for each group, yielding a sample size of 24. To account for attrition, 28 participants were recruited and randomly assigned to the experimental group or control group. The randomization procedure was performed by a third party using block randomization with a block size of 4.

### Intervention

Participants from both groups started the 6-week intervention 4 weeks after surgery. Those in the experimental group received two sessions of 60-min aquatic training, with three 60-min sessions of home exercises each week for 6 weeks, whereas those in the control group performed home exercises only (five 60-min sessions each week for 6 weeks). The aquatic pool was 7 m wide and 10 m long, with a graded depth from 0.9 to 1.3 m. The temperature of the pool was set at 34–36 °C, and the therapist and participant ratio was 1:2. A physiotherapist with 8 years of experience in aquatic therapy provided the aquatic training sessions. This intervention was administered from January 2015 to May 2016.

The aquatic exercise protocol consisted of 5 min of warm-up with stretching of the leg muscles and walking in water, 50 min of main exercises, and 5 min of cooldown stretching exercises (Online Appendix S1). The home exercise protocol consisted of general lumbar stability exercises for patients who had undergone lumbar fusion. The patients were provided a self-monitoring exercise checklist to help them adhere to and improve their exercise program.

### Outcome measures

The primary outcomes were pain intensity and disability, and the secondary outcomes were mobility, muscle strength, lumbopelvic stability, and LM muscle thickness. The measurement of secondary outcomes and evaluation of exercise intervention were performed by the first author (AH Huang), and the primary outcomes were evaluated by a physiotherapist with 5 years of working experience, who was blinded to the grouping of the participants.

Pain intensity was assessed using the Numerical Pain Rating Scale (NPRS). The NPRS is an 11-point scale from 0 to 10, with 0 indicating “pain free” and 10 indicating “the worst pain^39^”. Patients were asked to rate their resting pain experienced in the past week and before and after the intervention. The Chinese version of the modified ODI was used to assess the patients’ disability level^40^. The modified ODI consists of nine dimensions, namely pain intensity, personal care (e.g., washing, dressing), lifting, walking, sitting, standing, sleeping, social life, and traveling. Each category contains six questions representing different levels of problems and is scored from 0 to 5. The total score can be transformed into a percentile score from 0 to 100.

The Timed Up and Go Test (TUGT) was used to assess the patients’ mobility. This test starts with the patient seated on a chair. At the start signal, the patient is instructed to stand up and walk as fast as possible for 3 m, turn, walk back to the chair, and return to the seated position. The time was recorded by a handheld stopwatch. Two trials were performed, and the best recorded time was used for analysis^41^.

The isometric muscle strength of the trunk extensors and flexors was measured in the sitting position using a hand-held dynamometer (MicroFET2; HOGGAN, Atlanta, GA, USA)^5^. The pelvis was supported at the posterior superior iliac spine, and the lower limbs were supported at the knees and the ankles. A harness was attached to the shoulders and the chest just below the underarms, and it was mounted horizontally on a dynamometer. Two maximal efforts for both flexion and extension were performed, and the mean value was analyzed. Our results are expressed in pounds (lbs). This test had excellent between-week test–retest reliability, with the intraclass correlation coefficients (ICC (3,2)) between 0.95 and 0.96.

Hip movement range was used to indicate the level of control of the lumbopelvic stability during the one-leg loading test in the supine position^42^. The pressure biofeedback unit (PBU; Stabilizer; Chattanooga Group Inc., Hixson, TN, USA) was used to monitor the pressure changes under the lumbopelvic region during the one-leg loading test. Participants began with their hip and knee at 90° flexion, with the PBU positioned under the lumbar spine with a base pressure of 40 mmHg. The participants were then asked to perform an abdominal draw-in maneuver and maintain the PBU at a pressure of 45–55 mmHg whilst lowering one leg into extension. The test was stopped if the pressure of the PBU was outside the range of 45–55 mmHg, and the hip movement range was recorded. A broader range demonstrated a greater capacity for controlling lumbopelvic stability. The hip movement range was measured using a smartphone (Clinometer app; Plaincode, Stephanskirchen, Germany), which was tied to the thigh just above the femur lateral epicondyle using a smartphone armband. A camera (Panasonic LUMIX GX1, Japan; Full HD 1080i @ 60FPS AVCHD) recording was used to monitor the threshold when the PBU pressure was outside the target range of control. The test was performed thrice, and the averaged value was analyzed. This measurement had an excellent between-week test–retest reliability (ICC (3,3) = 0.99)^43^, which was established in our pilot study.

Rehabilitation ultrasound image measurement of the LM muscle thickness was conducted using a Philips HD11 XE ultrasound imaging apparatus with a 5–12-MHz linear transducer (Philips Medical Systems, Bothell, WA, USA). The participants were positioned in prone, with a pillow placed under the lower abdominals to minimize lumbar lordosis^22^. The muscles were imaged in the parasagittal section. The examiner was required to maintain the linear transducer in the plane of the facet joint and record the thickness of the multifidus muscle at the facet joint to the fascial line in the resting position as well as during hip extension with knee extension. LM muscle thickness was measured at five levels (L5/S1, L4/L5, L3/4, L2/L3, L2/L1), and the between-week test–retest reliability (ICCs (3,1)) was between 0.79 and 0.89 for the measurement at L2/L1 and between 0.84 and 0.92 for the measurement at other levels.

To determine the relative variation of individual muscle thickness, the thickness value obtained in each contraction task was subtracted from the value obtained when the LM muscle was in the resting position and then divided by the value in the resting position.

### Statistical analysis

Data were analyzed using SPSS (version 20 for Mac; SPSS Inc., Chicago, IL, USA). Independent *t* tests (age, height, weight and BMI) and chi-square tests (gender) were used to examine the between-group differences of the participants’ characteristics at baseline. Two-way ANOVA with repeated measures was used to compare the between-group differences before and after the interventions, with a significance level set at α = 0.05. If a significant interaction was identified, post hoc tests with a Bonferroni were used to assess the changes before and after the intervention, with the significance level set at 0.05. All analyses were conducted using the intention-to-treat approach. The missing data from those patients who had left the study during the intervention or before the post-test would be replaced using the pre-test data.

## Supplementary Information

Supplementary Information.

## Data Availability

The datasets generated during and/or analyzed during the current study are available from the corresponding author on reasonable request.
