# Supplementary material for: Effects of early aquatic exercise intervention on trunk strength and functional recovery of patients with lumbar fusion: a randomized controlled trial
Source: Sci Rep. 2023 Jul 3;13:10716. doi: 10.1038/s41598-023-37237-3 (PMC10317955; doi:10.1038/s41598-023-37237-3)
Supplement: Supplementary file 1 — Supplementary Information. [file 41598_2023_37237_MOESM1_ESM.docx]

|  | Aquatic exercise | |
| --- | --- | --- |
|  | Description | Photo |
| warm-up and cooldown | Standing in neutral position, facing the ledge, and place your hands on it. Lift one leg and position it on the side of the pool. Slowly lean your trunk forward while keeping your knee straight. Return to the starting position and repeat with the other leg.  Frequency: 10 sec/time, 10 time/set | 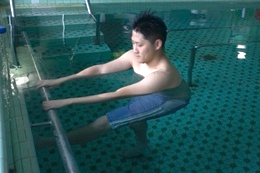 |
|  | Standing in neutral position with your back facing the edge. Bring your knee up towards your chest and grasp it with your opposite hand. Pull your leg across your body, as demonstrated. This will increase the stretch.  Frequency: 10 sec/time, 10 time/set | 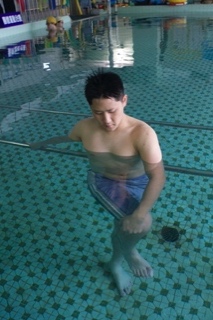 |
|  | Water walking (forward). Start by walking forward in chest-high water. Increase speed to make it more difficult.  Frequency: 5 time/set | 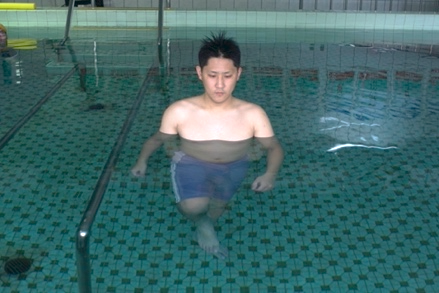 |
| main exercises | Standing in neutral position with feet shoulder width. Have arm by side with hold kickboard on hand. Bring arm pushes down and hold kickboard just below the water surface with slight squat.  Frequency: 10 sec/time, 10 time/set | 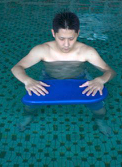 |
|  | Standing in neutral position with feet shoulder width. Have one side with hold kickboard on arm. Bring arm side pushes down and hold kickboard just below the water surface with slight squat.  Frequency: 10 sec/time, 10 time/set | 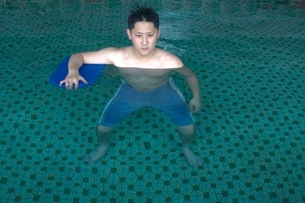 |
|  | Standing in neutral position with feet shoulder width. Begin to raise their bilateral arm forward and backward movement (shoulder flexion and extension at -20° to 20°), keep trunk erect.  Frequency: 10 sec/time, 10 time/set | 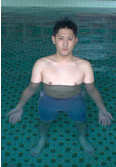 |
|  | Standing in neutral position with feet shoulder width. Begin to raise their one leg out to side (hip abduction at 0° to 45°), keep trunk erect.  Frequency: 10 sec/time, 10 time/set | 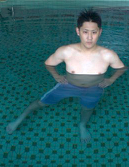 |
|  | Standing in neutral position with feet shoulder width. Begin to bilateral arm breaststroke movement with keep trunk balance in standing position. Frequency: 10 sec/time, 10 time/set | 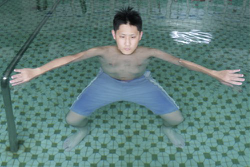 |
|  | Hold floating device in each hand and position in arm by side, raise bilateral knees to get into the 90-90 positions with keeping the body stable at 10 second, next movement to a prone position in water with keeping arm perpendicularly hold 10 second  Frequency: 10 sec/time, 10 time/set | 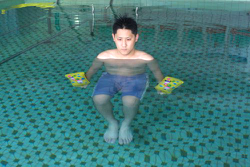 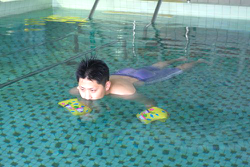 |

| Home exercise | |
| --- | --- |
| Description | Photo |
| Abdominal bracing  The subject was lying with feet flat on the floor with breath normally throughout, gently contract the abdominal muscles and try to keep the pelvic floor and lower abdominal muscles contracted together. Do this by pulling your umbilicus in towards your back.  Frequency**:**  10 time/set, 10 set, 5 times per week | 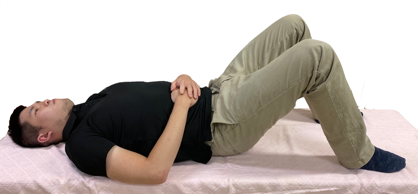 |
| Bridge exercise  The subject was lying on the floor with feet flat, hip-width apart, knees bent at 90°, toes facing forwards and your hands by sides, palms facing down. The subject lifts their buttocks to the body is in one straight line. Subjects performed the back bridge on the floor and kept their shoulders on the floor.  Frequency**:**  10 time/set, 10 set, 5 times per week | 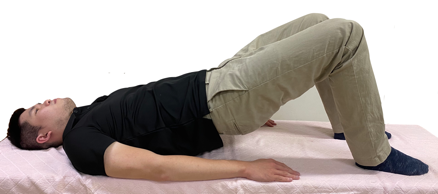 |
| Single one bridge exercise  Keep subject lifts buttocks and still as you slowly lift afoot. Only your feet should move. Your body should remain still.  Frequency**:**  10 time/set, 10 set, 5 times per week | 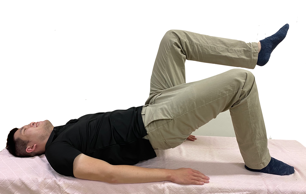 |
| HIP ABD  Stand with subject legs shoulder width and have a table or chair by your side for stability. maintain an abdominal bracing to protect the back from injury. Keep your leg straight out to the side of your body slowly.  Frequency**:**  10 time/set, 10 set, 5 times per week | 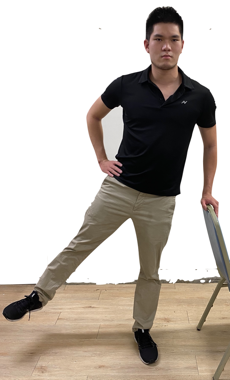 |
| Trunk training  The subject keeping the trunk in the same position, lift your knee upward and hold this position for 10 sec. Return to the starting position in a smooth movement, then switch sides and repeat the exercise.  Frequency**:**  10 time/set, 10 set, 5 times per week | 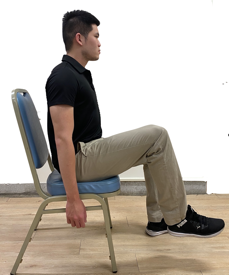 |
| Walk training  The subject maintains slowly walk 20 minutes of activity at least 5 days a week. | 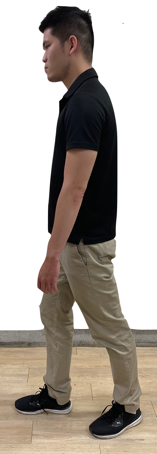 |

Appendix 1 Description of the aquatic and home exercises used in the present study.
